# Intranasal ondansetron microemulsion counteracting the adverse effects of cisplatin: animal study

**DOI:** 10.1007/s43440-022-00435-3

**Published:** 2022-12-14

**Authors:** Mai Mansour, Maha Nasr, Omar A. H. Ahmed-Farid, Rania F. Ahmed

**Affiliations:** 1 Department of Pharmaceutics and Industrial Pharmacy, Faculty of Pharmacy, Ain Shams University, African Organization Unity Street, Cairo, 11566 Egypt; 2 Department of Physiology, National Organization for Drug Control and Research, Giza, 12553 Egypt; 3 Department of Pharmacology, Medical Research and Clinical Studies Institute, National Research Centre (ID: 60014618), Dokki, Giza, 12622 Egypt

**Keywords:** Ondansetron, Microemulsion, Intranasal, Cisplatin, Pica

## Abstract

**Background:**

Cisplatin is considered one of the most effective and commonly used chemotherapeutic drugs, but despite its high therapeutic effectiveness, most patients treated with cisplatin suffer from nausea and vomiting, neurotoxic side effects, and cerebral psychiatric disorders such as depression. Therefore, the aim of the current work was to explore whether a selective 5-HT_3_ receptor antagonist (Ondansetron) administered via the oral route or intranasally in microemulsion form would alleviate cisplatin’s adverse effects.

**Methods:**

The selected ondansetron microemulsion was characterized in vitro for particle size, polydispersity, zeta potential, morphology, and nasal permeation, and in vivo in terms of anti-emetic and antidepressant activity, with the assessment of biochemical markers in brain homogenates.

**Results:**

Results revealed that both orally administered ondansetron and intranasally administered microemulsion were able to counteract the pica effect by increasing food consumption, water intake, and decreasing kaolin intake. They were also able to increase BDNF, normalize IL-6, increase serotonin, and normalize NOx, MDA, GSSH/GSH as well as 8OHdG levels in rats’ brain homogenates. The intranasal ondansetron microemulsion displayed superiority compared to oral conventional ondansetron in terms of increasing food intake, reduction of stomach content, and normalization of serotonin turnover.

**Conclusion:**

Ondansetron microemulsion can be administered by an alternative route of administration (intranasal) rather than oral, for patients on cisplatin chemotherapy.

**Graphical Abstract:**

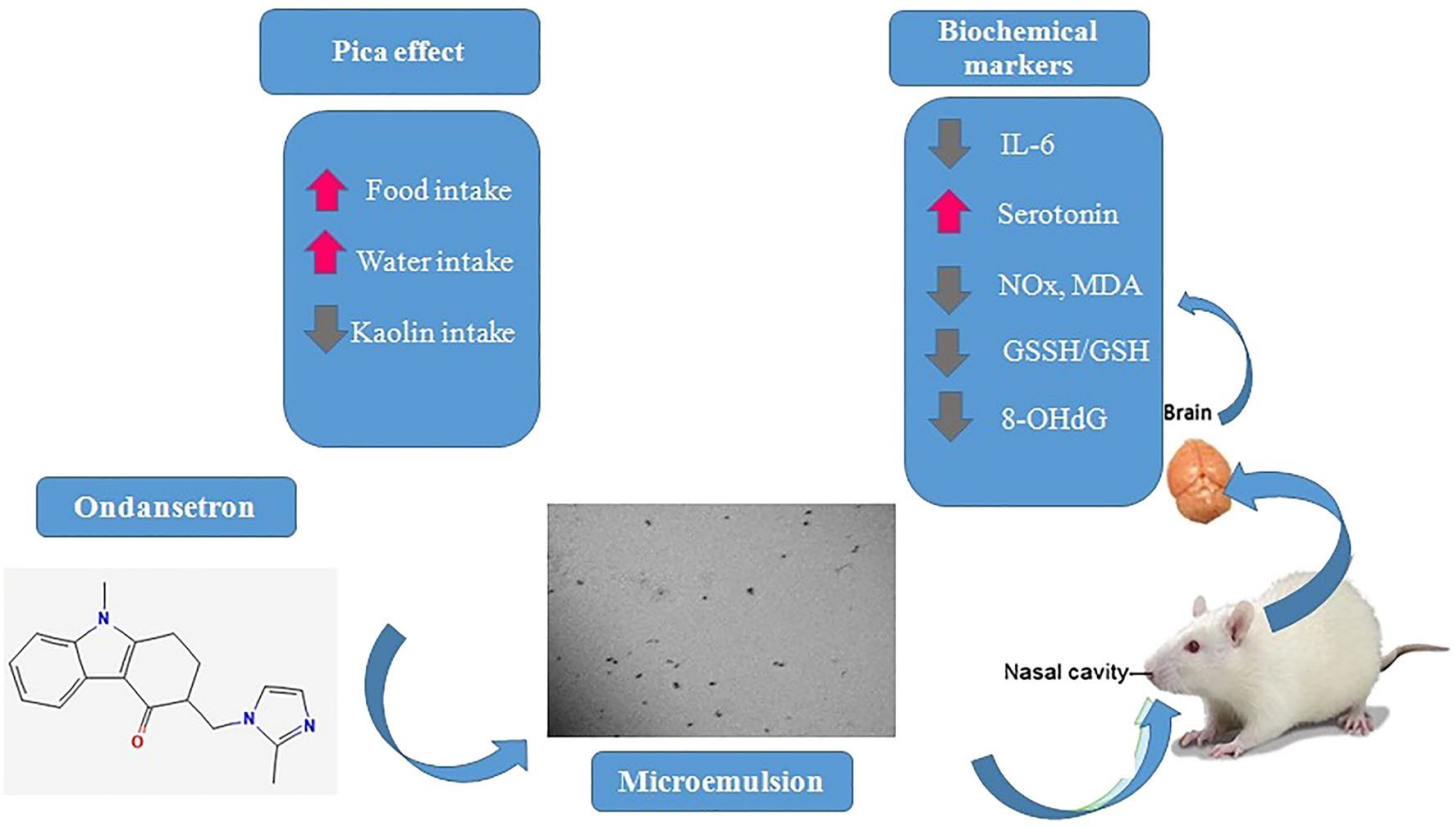

**Supplementary Information:**

The online version contains supplementary material available at 10.1007/s43440-022-00435-3.

## Introduction

Nausea and vomiting are reported to be two of the commonly reported adverse reactions of chemotherapy; extremely influencing the quality of patients’ lives and decreasing their compliance to treatment [1]. Usually, acute phases of chemotherapy-induced nausea and vomiting (CINV) occur within the first 24 h after chemotherapy, while delayed CINV occurs after 24 h and up to 120 h after chemotherapy [2, 3].

Cisplatin has long been stated as a main chemotherapeutic agent in the treatment protocol of multiple cancers, acting by inducing apoptosis in cancer cells through cross-linking DNA with the purine bases; resulting in impairing the cellular DNA repairing mechanisms and prompting DNA damage [4]. However, despite its high therapeutic effectiveness, most patients treated with cisplatin suffer from delayed nausea and vomiting [5]. In addition, cisplatin has also been reported to exhibit prominent neurotoxic side effects by elevating the reactive oxygen species (ROS) and increasing Platinum–DNA (PT–DNA) binding, with consequent neuronal apoptosis and inhibited neurogenesis, leading to neuronal loss and stimulation of neuroinflammation [6, 7]. Thus, patients may also experience neurotoxic side effects and cerebral psychiatric disorders such as depression and anxiety [8–10]. Therefore, it would be beneficial to concomitantly use an anti-emetic drug with a potential neuroprotective effect for patients treated with cisplatin [11].

Ondansetron is a selective 5-HT_3_ receptor antagonist which is used for prophylaxis and treatment of nausea and vomiting caused by chemotherapy and radiotherapy. In addition, ondansetron was found to reduce serotonin turnover and ameliorate symptoms of depression [12, 13], which makes it the optimum drug of choice for cisplatin-treated patients. However, ondansetron suffers from low oral bioavailability and first-pass metabolism [14, 15]; therefore, an alternative to the oral route can provide a solution to such a challenge. The intranasal route has long been used as a non-invasive mode of administration for several drugs designed for rapid permeation from the nasal mucosa to the systemic circulation and brain for treating central nervous system disorders, improving pharmacokinetics, and overcoming first-pass effect to enhance pharmacological activity [16, 17].

In addition to the selection of the route of administration, nanoparticles have been introduced as valuable means of enhancing the permeation of drugs across membranes, among which is the nasal membrane [18], as well as overcoming the pharmaceutical limitations of drugs and optimizing their therapeutic potential [19, 20]. Among the promising nanoparticles that can be used for such a purpose are microemulsions; which are basically nanoemulsions but are formed spontaneously by the combination of certain proportions of oils, surfactants and cosurfactants [21–24]. Microemulsions have been reported to permeate effectively across the nasal mucosa; bypassing the blood–brain barrier and delivering the drugs at adequate concentrations to the brain [25–27]. Therefore, the aim of the current work is to investigate the intranasal delivery of ondansetron in microemulsion form, the enhancement of the therapeutic potential of the drug as an anti-emetic, neuroprotective, and antidepressant agent, and its counteraction of the adverse effects of cisplatin treatment. To the current date, ondansetron has not been loaded in our reported microemulsion before for the same purpose, or any other purpose.

## Materials and methods

### Materials

Captex 500 (Triacetin) (102-76-1) was purchased from Abitec company, USA. Tween 20 (P1376), kaolin (K7375), gum arabic (G9752) and ethanol (34852-M) were purchased from Sigma Aldrich company, Germany. Ondansetron was purchased from Fraken pharmaceutical company, China.

### Preparation of ondansetron microemulsions

Captex 500, tween 20, and ethanol 95% were chosen as the oily phase, surfactant, and cosurfactant for the solubilization of ondansetron, respectively, based on a preliminary conducted solubility study (data not shown). The water dilution method was adopted for the formulation of microemulsions [28, 29], to construct two phase diagrams (ratio 1:1 and 2:1 surfactant:cosurfactant), in which the ratio of captex 500 to surfactant/cosurfactant mixture was varied between 1:9 and 9:1. Two microemulsion formulations (F1 and F2) were selected from the constructed diagrams (Table 1), to be loaded with 20 mg ondansetron followed by magnetic stirring till its solubilization; yielding a concentration of 0.2%. As shown in Table 1, F1 was composed of 2.5 g captex 500, 1.25 g tween 20, 1.25 g ethanol, and 5 ml water, while F2 was composed of 2.5 g captex 500, 1.67 g tween 20, 0.83 g ethanol and 5 ml water. The two formulations were kept at refrigeration temperature (4–8 °C) for further investigation.

**Table 1:** Composition and properties of the selected ondansetron microemulsion formulations

Code*	Composition (w/w%)				Particle size (nm) Z average diameter calculated by intensity percent		PDI		Zeta potential (mV)	
	Oil	Tween 20	Ethanol	Water						
					Fresh	After storage	Fresh	After storage	Fresh	After storage
F1	25%	12.5%	12.5%	50%	67.85 ± 25.35	72.22 ± 28.44	0.66 ± 0.23	0.67 ± 0.21	0.22 ± 0.01	0.20 ± 0.03
F2	25%	16.7%	8.3%	50%	13.06 ± 0.23	14.21 ± 0.33	0.45 ± 0.01	0.46 ± 0.01	0.18 ± 0.03	0.19 ± 0.02

*Each formulation contained 0.2% ondansetron

### Measurement of particle size, polydispersity index (PDI) and zeta potential of the selected ondansetron microemulsion formulations

The particle size, PDI and zeta potential of formulations F1 and F2 were measured using the Zetasizer device (Model ZS3600, Malvern, UK). The measurements were performed again after 3 months of storage, to assess the physical stability of the microemulsions.

### Ex vivo nasal permeation of the selected ondansetron microemulsion formulations

To assess the nasal permeation ability of formulations F1 and F2, their ex vivo diffusion across sheep nasal mucosa was tested [18]. The mucosa was cleaned, then placed in a Franz diffusion apparatus (VariomagTelesystem, Germany), using a cell of a diameter 1.77 cm^2^. The receptor medium was 7.5 ml phosphate buffer pH 7.4 containing 2% tween 20 to ensure sink conditions for ondansetron. An aliquot of 200 µl of either F1 or F2 was placed in the donor compartment, and samples were withdrawn from the receptor medium at certain time intervals (5 till 360 min) with replacement using fresh medium. The permeated ondansetron was quantified using HPLC (Agilent, USA) using C18 column (Agilent, USA), phosphate buffer pH 2.2: acetonitrile 73:27 as mobile phase at a flow rate 1 ml/min, and analysis at wavelength 246 nm [30].

### Morphology using transmission electron microscopy (TEM)

The selected microemulsion formulation was assessed for morphology using TEM, after negative staining with 2% uranyl acetate (JEM-100 S, Japan).

### In vivo study

Adult male Wistar albino rats (150–170 g) were purchased from the animal house of the national research centre (NRC). Upon arrival, the animals were acclimatized for 7 days before further proceeding with the experimental research. All experimental procedures were approved by the NRC ethical committee (approval No. 20/175).

### Measurement of kaolin consumption (pica effect)

It was reported that after cisplatin injection, rats tend to consume kaolin (indicating pica effect) [16], since rats respond to emesis-stimulating factors by induction of pica; which is the consumption of a substance without any nutritional value such as kaolin. Thus, pica is considered analogous to vomiting in species which are unable to vomit. Moreover, rats expressing the pica response were demonstrated to have increased gastric retention of solid material, thus, stomach content weight has been designated as an indicator for anti-emetic effect [16, 31].

Rats were randomly divided into four groups (eight rats each); group 1 was designated as normal control, group 2 was the cisplatin model group (Model), group 3 was treated with ondansetron in its conventional form as a suspension in distilled water (1.3 mg/kg) orally for 14 days [11], and group 4 received ondansetron microemulsion (1.3 mg/kg) intranasally for 14 days. Cisplatin (7.5 mg/kg; ip) [32] was injected on day 5 and day 10 for groups 2, 3 and 4. Pica test was performed on the last day after the administration of the last treatment. Forced swimming test (FST) was performed 24 h later to assess the behavioral antidepressant activity [33, 34], and rats were sacrificed 1 h after the forced swimming test by decapitation. Stomach content weights were determined to assess the anti-emetic effect [16, 31]. Brains were removed and homogenized in phosphate buffer for further procedures.

### Measured parameters in brain homogenates

Brain-derived neurotrophic factor BDNF and IL-6 were determined using commercial ELISA kits according to the manufacturer procedure (Glory Science Co., Ltd CAT. NO. 30714) and (Glory Science Co., Ltd CAT. NO. 31069), respectively. In addition, the following parameters were determined by HPLC (Agilent HP 1200, USA) by comparing samples to reference standards: serotonin (5-HT) and 5-Hydroxyindoleacetic acid (5-HIAA) as antidepressant parameters [35]; NOx (Nitrates + nitrites) [36], MDA level [37–39], and the ratio of thiols compounds of oxidized (GSSG) and reduced (GSH) glutathione [40, 41] as nitrosative and oxidative stress parameters; as well as 8-hydroxy-2-deoxyguanosine (8-OhdG) content [42] as DNA fragmentation parameter.

### Statistical analyses

Before proceeding statistical analysis, data values were checked for normality using Kolmogorov–Smirnov test. The data are presented as mean ± SE. Data were analyzed by one-way ANOVA followed by the Tukey–Kramer post hoc test using GraphPad Prism software (version 9, USA). The significance level was set to *p* < 0.05 for all statistical tests.

## Results

### Measurement of particle size, polydispersity index (PDI) and zeta potential of the selected ondansetron microemulsion formulations

Two formulations (F1 and F2) were selected from the pseudoternary diagrams, each was composed of 25% oil, 50% water, and ratio of surfactant: cosurfactant 1:1 and 2:1, respectively. As shown in Table 1, formulation F2 containing higher proportion of tween 20 displayed significantly smaller particle size and PDI than F1 (*p* < 0.05), and both displayed almost neutral charge. In addition, the microemulsions preserved their clarity and homogeneity, as well as their particle size, PDI and zeta potential when stored at refrigeration temperature for 3 months, as manifested by the non-significant change in their values (*p* > 0.05) (Table 1).

### Ex vivo nasal permeation of ondansetron microemulsion formulations

The HPLC method was used to quantify the amount of ondansetron permeated across sheep nasal mucosa (with a linearity range of 2.5–75 µg/ml, limit of detection 0.9 µg/ml, limit of quantification 2 µg/ml, % relative standard deviation for system/method/intermediate precision less than 2%, and mean accuracy 100.5%) [24]. The permeation of the microemulsion formulations across sheep nasal mucosa was assessed by the Franz diffusion apparatus, as detailed in the methodology section (Supplementary 1). As shown in Supplementary 1, formulation F2 displayed significantly higher cumulative percent released of ondansetron over a period of 6 h (*p* < 0.05), reaching 98.85%. Since formulation F2 displayed smaller particle size and PDI, as well as higher ex vivo permeation across sheep nasal mucosa compared to F1, F2 was the formulation of choice for further characterization.

### Morphology using transmission electron microscopy (TEM)

As shown in Supplementary 2, the microemulsion formulation F2 displayed a small particle size which was comparable to what was obtained with the Zetasizer device, with spherical morphology, complying with other authors [43].

### In vivo study

#### Effect of ondansetron and its microemulsion formulation on the immobility duration in the FST

**Fig. 1:**
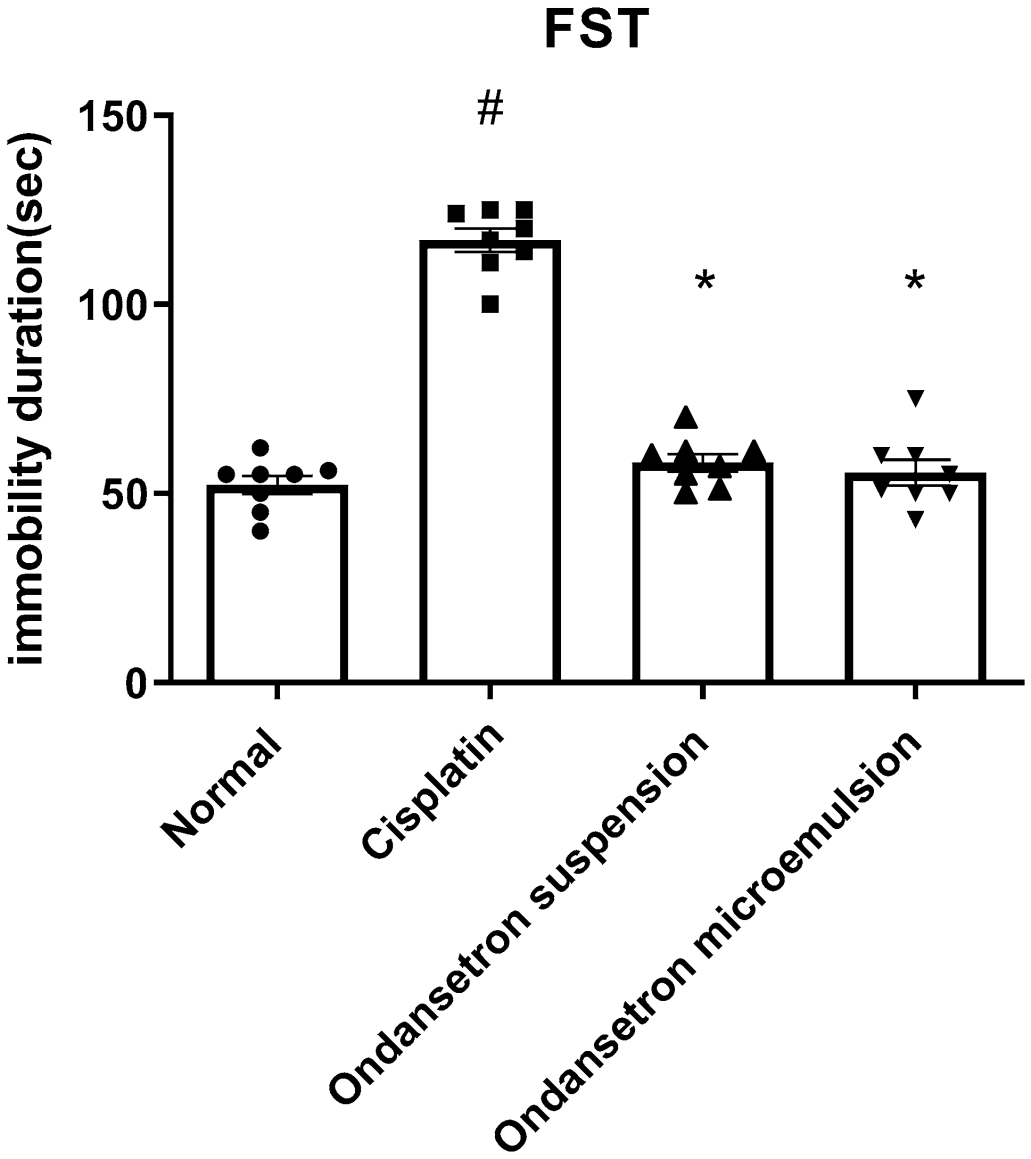
Effect of cisplatin, ondansetron suspension, and ondansetron microemulsion on the immobility duration in the forced swimming test (FST). Data were analyzed by One-way ANOVA followed by the Tukey–Kramer Post hoc test. The data are presented as mean ± SE. *N* = 8. ^#^Statistical significance from normal control, *Statistical significance from cisplatin group at *p* < 0.05

As shown in Fig. 1, cisplatin injection resulted in a significant increase (*F*_3, 28_ = 118.8, *p* < 0.0001) in the immobility duration in the FST by 123.9% at *p* < 0.0001 in comparison with the normal control. Oral ingestion of the conventional ondansetron suspension as well as intranasal administration of the ondansetron microemulsion formulation under investigation significantly reduced (*F*_3, 28_ = 118.8, *p* < 0.0001) the immobility duration in the FST by 50.3% and 52.6%, respectively, at *p* < 0.0001 compared to the cisplatin control.

### Effect of ondansetron and its microemulsion formulation on food and water consumption, kaolin intake, and stomach content

As shown in Table 2, cisplatin injection (7.5 mg/kg ip) significantly (*F*_3, 28_ = 229.5, *p* < 0.0001) reduced the food consumption by 70.2% at *p* < 0.0001, water consumption (*F*_3, 28_ = 139,8 *p* < 0.0001) by 55.2% at *p* < 0.0001 and significantly increased the kaolin intake (*F*_3, 28_ = 1049, *p* < 0.0001) to about 10 folds at *p* < 0.0001 and the stomach content weight (*F*_3, 28_ = 160.6, *p* < 0.0001) by 87.8% at *p* < 0.0001 compared to the normal control. Both ondansetron suspension administered orally and ondansetron microemulsion administered intranasally significantly increased (*F*_3, 28_ = 229.5, *p* < 0.0001) food consumption to 1.6 and 2.3 folds, respectively, at *p* < 0.0001, water consumption (*F*_3, 28_ = 139,8 *p* < 0.0001) by 71.3% and 76.6%, respectively, at *p* < 0.0001, decreased kaolin intake (*F*_3, 28_ = 1049, *p* < 0.0001) by 83.6% and 83.7%, respectively, at *p* < 0.0001 and stomach content weight (*F*_3, 28_ = 160.6, *p* < 0.0001) by 39% and 50.3% at *p* < 0.0001, respectively, compared to the cisplatin control. It is worth mentioning that the intranasal ondansetron microemulsion was superior to the conventional oral ondansetron in increasing food intake at *p* < 0.0001and reducing the stomach content of rats at *p* = 0.0419.

**Table 2:** Effect of cisplatin, ondansetron suspension and ondansetron microemulsion on the food and water consumption, kaolin intake and stomach content of rats

Groups	Food intake (g)		Water consumption (ml)		Kaolin intake (g)		Stomach content weight (g)
	Mean ± S.E		Mean ± S.E		Mean ± S.E		Mean ± S.E
	*N* = 8		*N* = 8		*N* = 8		*N* = 8
	Before induction	After induction	Before induction	After induction	Before induction	After induction	After decapitation
	*F*_3, 28_ = 0.7152, *p* = 0.5512	*F*_3, 28_ = 229.5, *p* < 0.0001	*F*_3, 28_ = 0.6950, *p* = 0.5629	*F*_3, 28_ = 139.8, *p* < 0.0001	*F*_3, 28_ = 0.009919, *p* = 0.9986	*F*_3, 28_ = 1049, *p* < 0.0001	*F*_3, 28_ = 160.6, *p* < 0.0001
Normal control	24.38 ± 0.66	28.13 ± 0.40	25.38 ± 0.32	26.25 ± 0.53	0.190 ± 0.014	0.186 ± 0.004	2.29 ± 0.16
Cisplatin control	24.75 ± 0.69	8.38 ± 0.40^#^	25.25 ± 0.45	11.75 ± 0.45^#^	0.191 ± 0.008	1.875 ± 0.045^#^	18.68 ± 0.37^#^
Ondansetron suspension	25.90 ± 0.84	13.44 ± 0.59^#^*	26.00 ± 0.33	20.13 ± 0.58^#^*	0.193 ± 0.01	0.308 ± 0.017^#^*	11.39 ± 0.89^#^*
Ondansetron microemulsion	25.38 ± 0.95	19.5 ± 0.77^#^*^@^	25.63 ± 0.46	20.75 ± 0.45^#^*	0.191 ± 0.008	0.306 ± 0.011^#^*	9.28 ± 0.41^#^*^@^

Data were analyzed by One-way ANOVA followed by the Tukey–Kramer Post hoc test

The data are presented as mean ± SE

*N* = 8 ^#^Statistical significance from normal control

*Statistical significance from cisplatin group

^@^Significantly different from ondansetron suspension at *p* < 0.05

### Effect of ondansetron and its microemulsion formulation on the brain level of BDNF

**Fig. 2:**
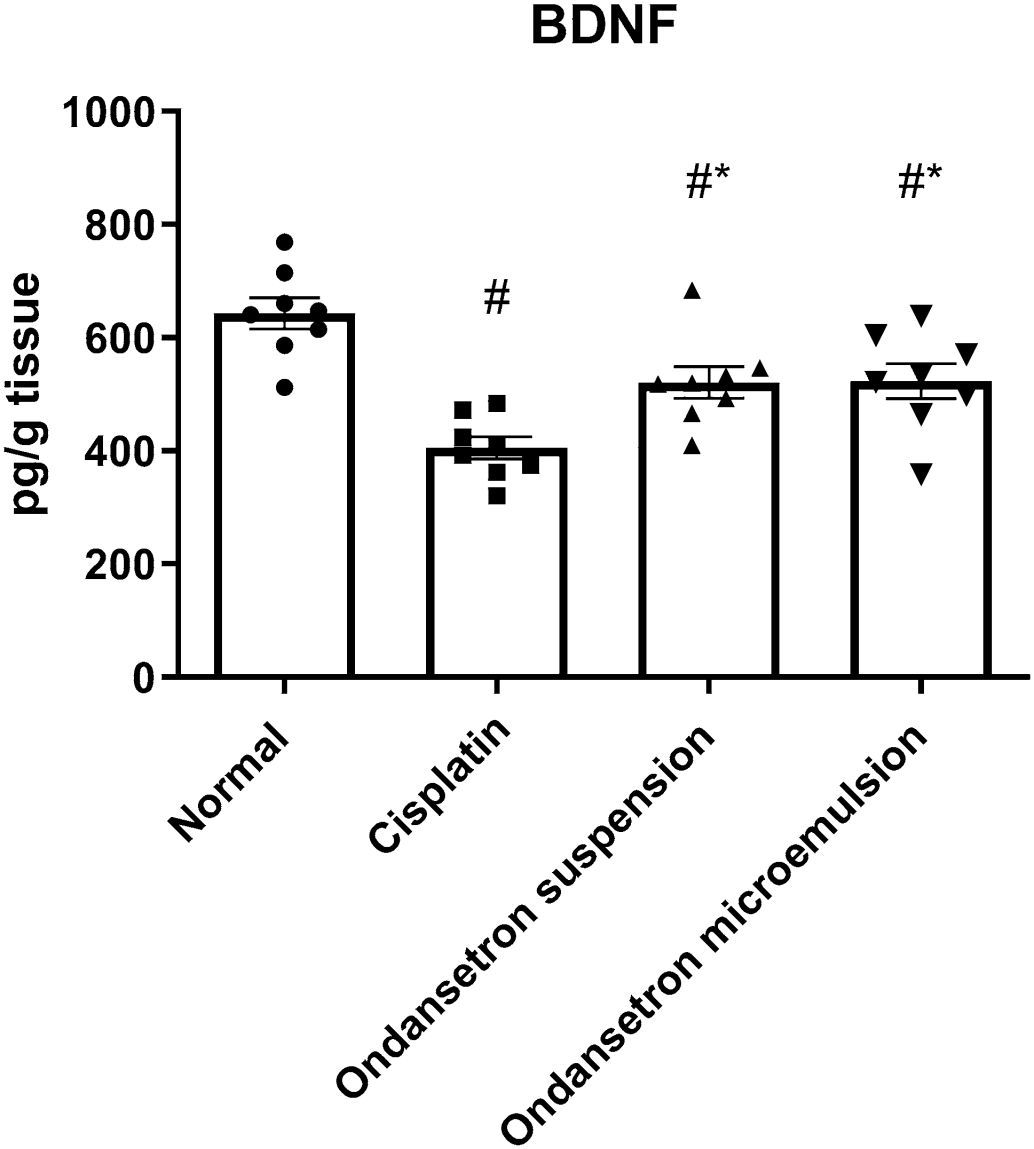
Effect of cisplatin, ondansetron suspension, and ondansetron microemulsion on brain levels of the brain-derived neurotrophic factor (BDNF). One-way ANOVA followed by the Tukey–Kramer post hoc test. The data are presented as mean ± SE. *N* = 8. ^#^Statistical significance from normal control, *Statistical significance from cisplatin group at *p* < 0.05

As shown in Fig. 2, cisplatin injection caused significant reduction (*F*_3, 28_ = 13.18, *p* < 0.0001) in the BDNF level by 37% at *p* < 0.0001 compared to the normal control. Oral ingestion of the conventional ondansetron suspension as well as intranasal administration of the ondansetron microemulsion significantly elevated (*F*_3, 28_ = 13.18, *p* < 0.0001) the BDNF level by 28.6% at *p* = 0.0236 and 29.1% at *p* = 0.0207, respectively, as compared to the cisplatin control.

### Effect of ondansetron and its microemulsion formulation on brain level of IL-6

**Fig. 3:**
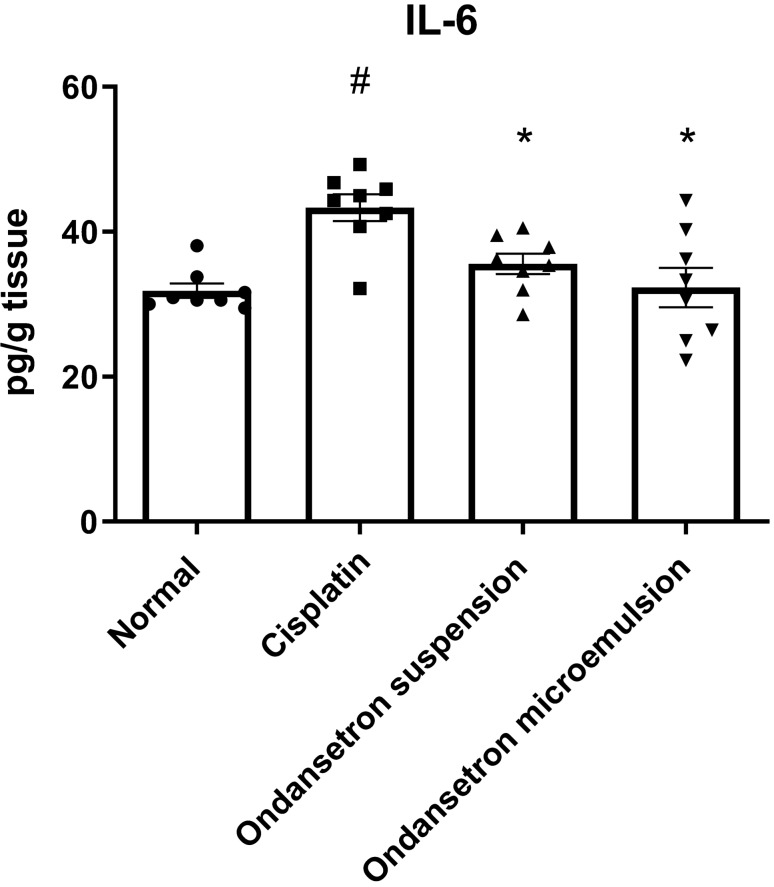
Effect of cisplatin, ondansetron suspension and ondansetron microemulsion on brain levels of interleukin-6 (IL-6). One-way ANOVA followed by the Tukey–Kramer post hoc test. The data are presented as mean ± SE. *N* = 8.^#^Statistical significance from normal control, *Statistical significance from cisplatin group at *p* < 0.05

Cisplatin injection caused significant increase (*F*_3, 28_ = 8.193, *p* = 0.0005) in the IL-6 level by 35.9% at *p* = 0.0008 compared to the normal control (Fig. 3). On the other hand, the oral ingestion of ondansetron suspension as well as the intranasal administration of the ondansetron microemulsion normalized the IL-6 level.

### Effect of ondansetron and its microemulsion formulation on brain serotonin level

**Fig. 4:**
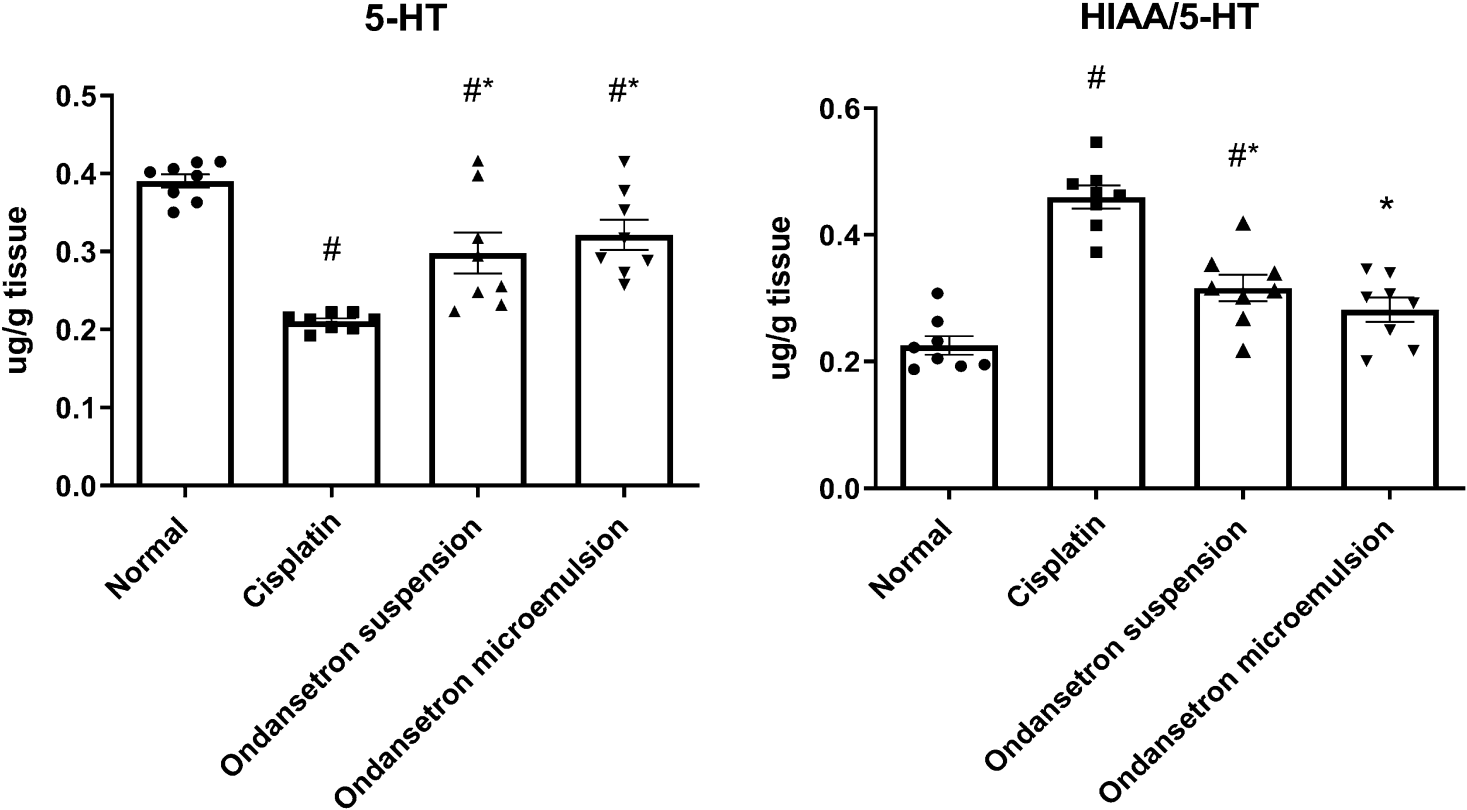
Effect of cisplatin, ondansetron suspension, and ondansetron microemulsion on brain levels of serotonin and serotonin turnover. One-way ANOVA followed by the Tukey–Kramer ost hoc test. The data are presented as mean ± SE. *N* = 8. ^#^Statistical significance from normal control, *Statistical significance from cisplatin group at *p* < 0.05. Serotonin (5-HT), 5-Hydroxyindoleacetic acid (5-HIAA), serotonin turnover ratio (5-HIAA)/(5-HT)

As shown in Fig. 4, cisplatin injection significantly reduced (*F*_3, 28_ = 19.05, *p* < 0.0001) the brain 5-HT level by 46.1% at *p* < 0.0001 and increased its turnover (*F*_3, 28_ = 29.49, *p* < 0.0001) represented by the increase in the HIAA/5-HT ratio by 103.8% at *p* < 0.0001. The oral ingestion of the ondansetron suspension as well as the intranasally administered ondansetron microemulsion significantly elevated (*F*_3, 28_ = 19.05, *p* < 0.0001) the 5-HT level by 41.7% at *p* = 0.0057 and 52.9% at *p* = 0.0004 as compared to the cisplatin control. Oral ingestion of the ondansetron suspension significantly decreased (*F*_3, 28_ = 29.49, *p* < 0.0001) the serotonin turnover by 31.2% at *p* < 0.0001. Meanwhile, the microemulsion formulation under investigation normalized the serotonin turnover.

### Effect of ondansetron and its microemulsion formulation on the cellular nitrosative and oxidative stresses parameters

**Fig. 5:**
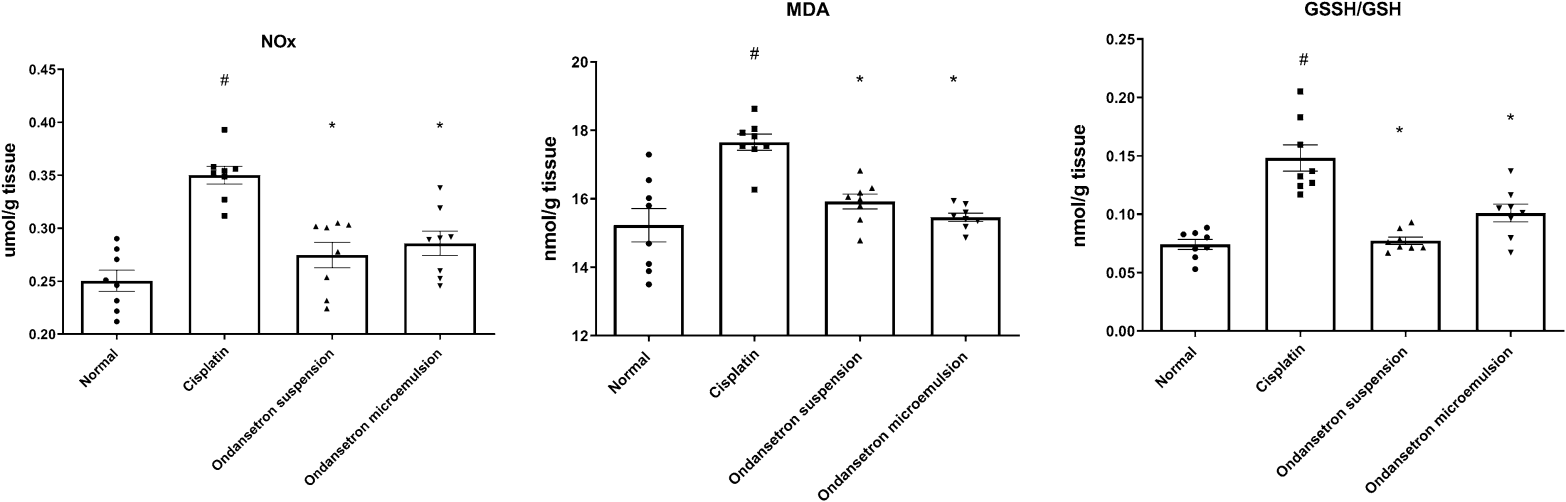
Effect of cisplatin, ondansetron suspension, and ondansetron microemulsion on cellular nitrosative and oxidative stress parameters. One-way ANOVA followed by the Tukey–Kramer post hoc test. The data are presented as mean ± SE. *N* = 8 ^#^Statistical significance from normal control, *Statistical significance from cisplatin group at *p* < 0.05. Malondialdehyde (MDA), NOx (Nitrates + nitrites), Oxidized glutathione (GSSG), reduced (GSH) glutathione

As demonstrated in Fig. 5, cisplatin injection resulted in a significant increase (*F*_3, 28_ = 16.34, *p* < 0.0001) in the brain NOx level by 39.9% at *p* < 0.0001 as well as a significant increase (*F*_3, 28_ = 13.52, *p* < 0.0001) in the MDA level by 16% at *p* < 0.0001 in comparison with the normal control. In addition, cisplatin injection shifted (*F*_3, 28_ = 22.32, *p* < 0.0001) the ratio of oxidized to reduced glutathione toward the oxidized state thus increasing the ratio by 99.8% at *p* < 0.0001 compared to the normal control. Interestingly, the oral ingestion of the ondansetron suspension as well as the intranasal administration of the ondansetron microemulsion formulation normalized the NOx level as well as the MDA level and shifted the ratio of GSSG/GSH was back to the normal state.

### Effect of ondansetron and its microemulsion formulation on cellular DNA fragmentation

**Fig. 6:**
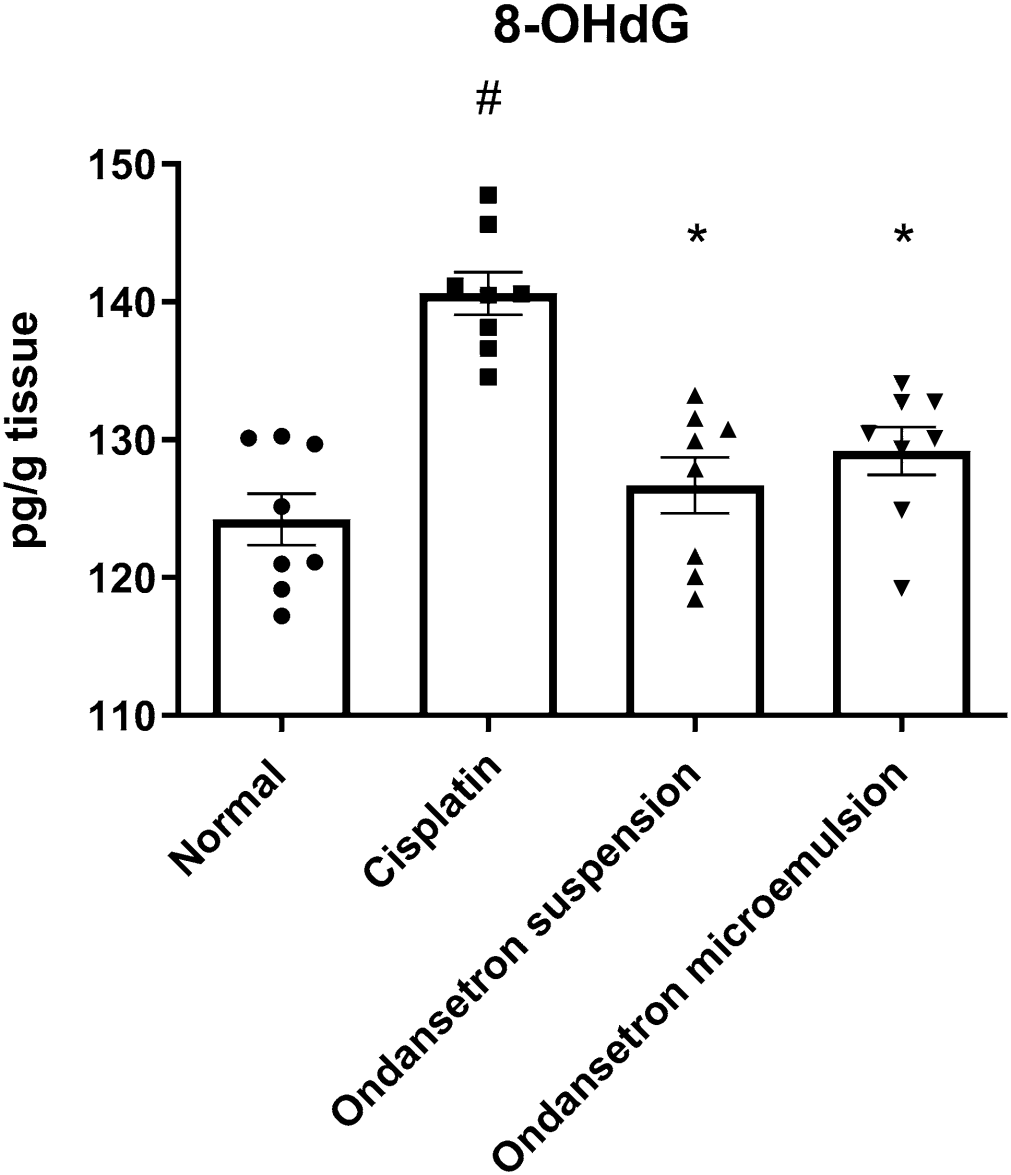
Effect of cisplatin, ondansetron suspension, and ondansetron microemulsion on brain 8-hydroxy-2-deoxyguanosine (8OHdG) levels. One-way ANOVA followed by the Tukey–Kramer post hoc test. The data are presented as mean ± SE. *N* = 8.^#^Statistical significance from normal control, *Statistical significance from cisplatin group at *p* < 0.05

As shown in Fig. 6, cisplatin injection significantly increased (*F*_3, 28_ = 16.03, *p* < 0.0001) brain cellular DNA fragmentation, represented by the increase in the 8OHdG level by 13.2% at *p* < 0.0001, in comparison with the normal control. The oral ingestion of the ondansetron suspension as well as the intranasal administration of the ondansetron microemulsion normalized the 8OHdG levels, indicating DNA preservation potential.

## Discussion

The formation of a homogenous nanosized dispersion is considered a cornerstone in formulating an efficient nanoparticulate system, especially for nasal delivery [44]. In the present study, two microemulsion formulations (F1 and F2) were tested as delivery carriers for ondansetron. Both formulations exhibited favorable physicochemical properties, manifested by small particle size, stability on storage, as well as ability to permeate across the nasal mucosa. However, formulation F2 was superior to F1, since it exhibited significantly smaller particle size, PDI, as well as twofold higher permeation of ondansetron across the nasal mucosa and could be attributed to its higher tween 20 content, which is reported to effectively incorporate the oily phase into the microemulsion leading to smaller particle size. Owing to its tensioactive traits, tween 20 can also effectively decrease the mucus viscosity and allow fluidization of the nasal mucosal membrane [18]. Formulation F2 was then compared in an in vivo study to the conventional ondansetron powder regarding its anti-emetic ability as well as neuroprotective and possibly antidepressant potential.

In the present study, cisplatin resulted in increased immobility duration in the FST along with a pronounced increase in the pica effect and the stomach content weight. BDNF and serotonin (5-HT) levels were decreased, IL-6, NOx, and MDA levels were elevated, and the GSSG/GSH ratio was increased indicating the existence of inflammation and pronounced nitrosative and oxidative stress status. Finally, 8-hydroxy guanosine (8OHdG) was increased, indicating cellular DNA damage.

Results of our study revealed that both orally administered suspension and intranasal microemulsion of ondansetron counteracted the adverse effects of cisplatin, as depicted by normalization of the immobility duration in the forced swimming test, reduction of the pica effect, the elevation of BDNF and normalization of IL-6 levels, the elevation of serotonin level, and normalization of oxidative and nitrosative parameters and 8OHdG levels. The intranasally administered ondansetron microemulsion exhibited superiority compared to the conventional suspension in terms of increased food intake, decreased stomach content, and normalization of the serotonin turnover.

Previous studies outlined the ability of ondansetron to reduce the pica effect in rats and antagonize the emetic effect of cisplatin [11]. Moreover, it was formerly reported to decrease the immobility duration time in the forced swimming test, increase serotonin (5-HT) and BDNF levels, and ameliorate symptoms of depression probably due to its ability to block the postsynaptic 5-HT_3_ receptors, resulting in a decrease in the 5-HT turnover rate [11–13]. Serotonin (5-Hydroxytryptamine; 5-HT) is a neurotransmitter that exhibits both physiological and psychological roles. It is mainly involved in the regulation of gastrointestinal motility, and it is one of the main neurotransmitters counteracting neurodegeneration and depression [45]. 5-HT_3_ receptor (5-HT_3_R) antagonists have long been used clinically to treat nausea and vomiting in patients receiving chemotherapy [46]. Previous studies were also concerned with the therapeutic ability of 5-HT_3_R antagonists in other diseases such as psychiatric disorders and neurodegenerative diseases. It was postulated that 5-HT_3_R antagonism potentiated the increase in extracellular 5-HT [47]. Moreover, the brain-derived neurotrophic factors (BDNF) which regulate the synaptic plasticity have a major role in the pathophysiology of depression and the effect of antidepressant treatments [48]. BDNF is co-localized on the serotonergic neurons, and it was found that expression of BDNF at least in part is regulated by 5-HT, where increased 5-HT levels improve the expression of the BDNF gene. On the other hand, BDNF promotes the survival and differentiation of 5-HT neurons. Thus, both BDNF and 5-HT are implicated in regulating neuronal survival and synaptogenesis [49].

In addition, it was previously reported that 8-hydroxy-2-deoxyguanosine (8OHdG); which is a repair product of oxidized guanine lesions, is directly connected to elevated oxidative stress or disease states, and could be used as a reliable marker of cellular DNA damage and repair [50–53].

Our results suggests that nanoformulations administered via an alternative route (intranasal rather than oral) could be a promising treatment modality for patients on chemotherapy, who are suffering from nausea and vomiting and cannot use antiemetics by the oral route. However, this study has two limitations which need to be addressed in futuristic studies: namely the pharmacokinetic data of ondansetron, and the impact of treatment on concomitant cisplatin/ondansetron-induced nephrotoxicity. Cisplatin is excreted in the urine during the first 24 h after administration and thus, it could cause serious damage on the proximal tubule cells of kidney if not quickly eliminated. It has been reported that the organic cation transporter 2 (OCT2) as well as multidrug and toxin extrusion proteins (MATEs) possess a vital role in eliminating cisplatin in renal proximal tubules. 5-HT3 antagonists such as ondansetron are known to be inhibitors of OCT2 and MATE1 and, therefore, could act as risk factors in cisplatin-induced nephrotoxicity. Previous preclinical investigations regarding this issue are controversial as it was formerly demonstrated by Badary et al. 2000, that single administration of ondansetron (0.2 mg/kg, ip) 1 h before injecting cisplatin (7 mg/kg, ip) did not intensify cisplatin-induced nephrotoxicity in mice and there was no significance observed regarding serum blood urea nitrogen or creatinine levels [54]. Similarly, Zirak et al. (2014) reported that administration of ondansetron in mice at a dose of 3 mg/kg ip twice a day for 3 days after single cisplatin injection at a dose of 20 mg/kg ip on day 1 showed no significance from the cisplatin control regarding both serum blood urea nitrogen and creatinine levels. Besides there was no significance regarding the pathological alterations in the kidney [55]. On the other hand, Li et al. (2013) reported that acute single injection of ondansetron at a dose of 6.4 mg/kg ip in mice, 30 min before injecting cisplatin at a dose of 10 mg/kg ip elevated both serum blood urea nitrogen and creatinine levels compared to the group administered cisplatin alone. In addition, aggravation in the histopathological alterations in the kidney was observed [56]. The difference between those studies could be attributed to the doses applied as well as the duration of each study. Meanwhile, regarding the clinical retrospective studies, it was reported by Kou et al. (2018) that co-administration of ondansetron with cisplatin could develop nephrotoxicity in cancer patients evaluated through increased serum creatinine levels and that higher cisplatin dosage with regular use of ondansetron could elevate the incidence of nephrotoxicity [57]. In general, nose to brain delivery is the simpler and direct way for brain targeting, which avoids bloodstream clearance due to the unique connection provided by the olfactory and trigeminal nerves between the brain and external environments. Following intranasal administration, drugs are exposed to the nasal mucosa, which is innervated by olfactory and trigeminal nerves; therefore, in the case of intranasal administration, three transport pathways are involved: the olfactory pathway in which the drug passes through the olfactory epithelium (paracellularly and extracellularly) into the olfactory bulb and further into the brain tissue or into the cerebrospinal fluid, the trigeminal pathway, in which the drug is transported via the nervous system, and the systemic pathway, in which the drug is absorbed across the nasal cavity into the systemic circulation and then across the blood brain barrier into the brain. The olfactory pathway as well the trigeminal pathway involve direct drug delivery to the CNS thus, one of the main advantages of using intranasal drug is the reduced risk of systemic adverse effects including hepato- and nephrotoxicity [58–62]. Hence, futuristic preclinical studies in our laboratory will focus on the evaluation of the risk benefit ratio of long-term application of the ondansetron microemulsion for medicinal use regarding the effectiveness *vs.* exaggeration of nephrotoxicity when used with cisplatin, and whether it would exhibit a better safety profile compared to the conventional orally administered drug.

From a pharmacokinetic perspective, as mentioned above, the intranasal delivery of ondansetron provides a route of its diffusion across nasal mucosa (in the olfactory region of the nasal cavity), which has direct access to brain areas. Moreover, it was reported that nanoparticles exhibit faster diffusion across nasal mucosa owing to their nanosize and better targeting potential in brain areas with improved concentrations of drug in brain. Our results displayed that the intranasal administration of ondansetron microemulsion was superior to the orally administered ondansetron suspension, but this observation needs to be verified using pharmacokinetic data by analyzing blood and brain tissue samples at different time intervals, to calculate peak concentration (Cmax), time to reach peak concentration (Tmax), and the area under the concentration–time curve (AUC). Moreover, the relative bioavailability for intranasal samples compared to their corresponding oral samples, the intranasal drug targeting efficiency (DTE%), and the nose to brain direct transport percentage (DTP%) by excluding the contribution of the systemic circulation through the blood brain barrier need also to be calculated.

## Conclusion

Ondansetron was proven to counteract the emetic, depressant, and neurotoxic effects of cisplatin, either in the oral conventional form or the intranasally administered microemulsion form, with the latter proven superior to the former in some parameters such as increased food intake and normalization of serotonin turnover. This provides an opportunity for ondansetron microemulsion to be administered by an alternative route of administration (intranasal) rather than oral, for patients on cisplatin chemotherapy. Future research work will include the comparative assessment of ondansetron pharmacokinetics in solution and microemulsion form, after administration using oral and intranasal routes. In addition, further in vivo studies will be conducted, to evaluate the effect of long-term application of the microemulsion formulation on the effectiveness and toxicity to all body organs (nephrotoxicity in particular), when used with cisplatin.

## Supplementary Information

Below is the link to the electronic supplementary material.

Supplementary file1 (JPG 126 KB)

Supplementary file2 (DOCX 1206 KB)

Supplementary file3 (XLSX 66 KB)

## Data Availability

All data generated or analyzed during this study are included in this published article and its supplementary information files.

## References

[1] Navari RM (2015). 5-HT3 receptors as important mediators of nausea and vomiting due to chemotherapy. Biochim Biophys Acta.

[2] Natale JJ (2018). Overview of the prevention and management of CINV. Am J Manag Care.

[3] Rashad N, Abdel-Rahman O (2017). Differential clinical pharmacology of rolapitant in delayed chemotherapy-induced nausea and vomiting (CINV). Drug Des Devel Ther.

[4] Ghosh S (2019). Cisplatin: the first metal based anticancer drug. Bioorg Chem.

[5] Li X, Qin Y, Liu W, Zhou XY, Li YN, Wang LY (2018). Efficacy of ginger in ameliorating acute and delayed chemotherapy-induced nausea and vomiting among patients with lung cancer receiving cisplatin-based regimens: a randomized controlled trial. Integr Cancer Ther.

[6] Chen C, Zhang H, Xu H, Zheng Y, Wu T, Lian Y (2019). Ginsenoside Rb1 ameliorates cisplatin-induced learning and memory impairments. J Ginseng Res.

[7] Ta LE, Espeset L, Podratz J, Windebank AJ (2006). Neurotoxicity of oxaliplatin and cisplatin for dorsal root ganglion neurons correlates with platinum-DNA binding. Neurotoxicology.

[8] Abdelkader NF, Saad MA, Abdelsalam RM (2017). Neuroprotective effect of nebivolol against cisplatin-associated depressive-like behavior in rats. J Neurochem.

[9] Almutairi MM, Alanazi WA, Alshammari MA, Alotaibi MR, Alhoshani AR, Al-Rejaie SS (2017). Neuro-protective effect of rutin against cisplatin-induced neurotoxic rat model. BMC Complement Altern Med.

[10] Oz M, Nurullahoglu Atalik KE, Yerlikaya FH, Demir EA (2015). Curcumin alleviates cisplatin-induced learning and memory impairments. Neurobiol Learn Mem.

[11] Feng X, Cheng Q, Meng Q, Yang Y, Nie K (2019). Effects of ondansetron and [6]-gingerol on pica and gut microbiota in rats treated with cisplatin. Drug Des Devel Ther.

[12] Gupta D, Radhakrishnan M, Kurhe Y (2014). Ondansetron, a 5HT3 receptor antagonist reverses depression and anxiety-like behavior in streptozotocin-induced diabetic mice: possible implication of serotonergic system. Eur J Pharmacol.

[13] Johnson BA, Ait-Daoud N, Ma JZ, Wang Y (2003). Ondansetron reduces mood disturbance among biologically predisposed, alcohol-dependent individuals. Alcohol Clin Exp Res.

[14] Roila F, Del Favero A (1995). Ondansetron clinical pharmacokinetics. Clin Pharmacokinet.

[15] Saynor DA, Dixon CM (1989). The metabolism of ondansetron. Eur J Cancer Clin Oncol.

[16] Barakat SS, Nasr M, Ahmed RF, Badawy SS, Mansour S (2017). Intranasally administered in situ gelling nanocomposite system of dimenhydrinate: preparation, characterization and pharmacodynamic applicability in chemotherapy induced emesis model. Sci Rep.

[17] Liu YL, Malik N, Sanger GJ, Friedman MI, Andrews PL (2005). Pica–a model of nausea? Species differences in response to cisplatin. Physiol Behav.

[18] Nasr M, Wahdan SA (2019). Neuroprotective effects of novel nanosystems simultaneously loaded with vinpocetine and piracetam after intranasal administration. Life Sci.

[19] Hatem S, Nasr M, Elkheshen SA, Geneidi AS (2018). Recent advances in antioxidant cosmeceutical topical delivery. Curr Drug Deliv.

[20] Amer SS, Nasr M, Mamdouh W, Sammour O (2019). Insights on the use of nanocarriers for acne alleviation. Curr Drug Deliv.

[21] Ramez SA, Soliman MM, Fadel M, Nour El-Deen F, Nasr M, Youness ER (2018). Novel methotrexate soft nanocarrier/fractional erbium YAG laser combination for clinical treatment of plaque psoriasis. Artif Cells Nanomed Biotechnol.

[22] Mohamed MA, Nasr M, Elkhatib WF, Eltayeb WN, Elshamy AA, El-Sayyad GS (2021). Nanobiotic formulations as promising advances for combating MRSA resistance: susceptibilities and post-antibiotic effects of clindamycin, doxycycline, and linezolid. RSC Adv.

[23] Mohamed MA, Nasr M, Elkhatib WF, Eltayeb WN (2018). In vitro evaluation of antimicrobial activity and cytotoxicity of different nanobiotics targeting multidrug resistant and biofilm forming Staphylococci. Biomed Res Int.

[24] Abu-Azzam O, Nasr M (2020). In vitro anti-inflammatory potential of phloretin microemulsion as a new formulation for prospective treatment of vaginitis. Pharm Dev Technol.

[25] Pires PC, Fazendeiro AC, Rodrigues M, Alves G, Santos AO (2021). Nose-to-brain delivery of phenytoin and its hydrophilic prodrug fosphenytoin combined in a microemulsion - formulation development and in vivo pharmacokinetics. Eur J Pharm Sci.

[26] Wen MM, Ismail NIK, Nasra MMA, El-Kamel AH (2021). Repurposing ibuprofen-loaded microemulsion for the management of Alzheimer’s disease: evidence of potential intranasal brain targeting. Drug Deliv.

[27] Froelich A, Osmałek T, Jadach B, Puri V, Michniak-Kohn B (2021). Microemulsion-based media in nose-to-brain drug delivery. Pharmaceutics.

[28] El-Gogary RI, Ragai MH, Moftah N, Nasr M (2021). Oleuropein as a novel topical antipsoriatic nutraceutical: formulation in microemulsion nanocarrier and exploratory clinical appraisal. Expert Opin Drug Deliv.

[29] Nasr M, Abdel-Hamid S, Moftah NH, Fadel M, Alyoussef AA (2017). Jojoba oil soft colloidal nanocarrier of a synthetic retinoid: preparation, characterization and clinical efficacy in psoriatic patients. Curr Drug Deliv.

[30] Basha M, Praveena B, Srinidhi M, Rahaman SK (2013). Method development and validation of ondansetronin bulk and pharmaceutical dosage form by stability-indicating RP-HPLC method. Int J PharmTech Res.

[31] Batra VR, Schrott LM (2011). Acute oxycodone induces the pro-emetic pica response in rats. J Pharmacol Exp Ther.

[32] Kumburovic I, Selakovic D, Juric T, Jovicic N, Mihailovic V, Stankovic JK (2020). Antioxidant effects of *Satureja hortensis* L. attenuate the anxiogenic effect of cisplatin in rats. Oxid Med Cell Longev.

[33] Ahmed RF, Abdel-Rahman RF, Farid OAHA, El-Marasy SA, Hessin AF (2014). Combined hepatoprotective and antidepressant effects of resveratrol in an acute model of depression. Bull Fac Pharm Cairo Univ.

[34] Porsolt RD, Le Pichon M, Jalfre M (1977). Depression: a new animal model sensitive to antidepressant treatments. Nature.

[35] Pagel P, Blome J, Wolf HU (2000). High-performance liquid chromatographic separation and measurement of various biogenic compounds possibly involved in the pathomechanism of Parkinson’s disease. J Chromatogr B Biomed Sci Appl.

[36] Papadoyannis IN, Samanidou VF, Nitsos CC (1999). Simultaneous determination of nitrite and nitrate in drinking water and human serum by high performance anion-exchange chromatography and uv detection. J Liq Chromatogr Relat Technol.

[37] Karatas F, Karatepe M, Baysar A (2002). Determination of free malondialdehyde in human serum by high-performance liquid chromatography. Anal Biochem.

[38] Karatepe M (2004). Simultaneous determination of ascorbic acid and free malondialdehyde in human serum by HPLC–UV. LCGC Asia Pacific.

[39] Lazzarino G, Di Pierro D, Tavazzi B, Cerroni L, Giardina B (1991). Simultaneous separation of malondialdehyde, ascorbic acid, and adenine nucleotide derivatives from biological samples by ion-pairing high-performance liquid chromatography. Anal Biochem.

[40] Jayatilleke E, Shaw S (1993). A high-performance liquid chromatographic assay for reduced and oxidized glutathione in biological samples. Anal Biochem.

[41] Yoshida T (1996). Determination of reduced and oxidized glutathione in erythrocytes by high-performance liquid chromatography with ultraviolet absorbance detection. J Chromatogr B Biomed Appl.

[42] Lodovici M, Casalini C, Briani C, Dolara P (1997). Oxidative liver DNA damage in rats treated with pesticide mixtures. Toxicology.

[43] Al-Karaki R, Awadallah A, Tawfeek HM, Nasr M (2020). Preparation, characterization and cytotoxic activity of new oleuropein microemulsion against HCT-116 colon cancer cells. Pharm Chem J.

[44] Mansour M, Elmowafy E, Gad HA (2021). Intranasal versus intraperitoneal Myrj 59-stabilized cubosomes: a potential armamentarium of effective anti-diabetic therapy. Coll Surf B Biointerfaces.

[45] Zulkifli MH, Viswenaden P, Jasamai M, Azmi N, Yaakob NS (2019). Potential roles of 5-HT3 receptor (5-HT3R) antagonists in modulating the effects of nicotine. Biomed Pharmacother.

[46] Yokoe T, Hayashida T, Nagayama A, Nakashoji A, Maeda H, Seki T (2019). Effectiveness of antiemetic regimens for highly emetogenic chemotherapy-induced nausea and vomiting: a systematic review and network meta-analysis. Oncologist.

[47] Sanchez C, Asin KE, Artigas F (2015). Vortioxetine, a novel antidepressant with multimodal activity: review of preclinical and clinical data. Pharmacol Ther.

[48] Bétry C, Etiévant A, Oosterhof C, Ebert B, Sanchez C, Haddjeri N (2011). Role of 5-HT(3) receptors in the antidepressant response. Pharmaceuticals.

[49] Martinowich K, Lu B (2008). Interaction between BDNF and serotonin: role in mood disorders. Neuropsychopharmacology.

[50] Abd-Allah H, Nasr M, Ahmed-Farid OAH, El-Marasy SA, Bakeer RM, Ahmed RF (2021). Biological and pharmacological characterization of ascorbic acid and nicotinamide chitosan nanoparticles against insulin-resistance-induced cognitive defects: a comparative study. ACS Omega.

[51] Abe T, Tohgi H, Isobe C, Murata T, Sato C (2002). Remarkable increase in the concentration of 8-hydroxyguanosine in cerebrospinal fluid from patients with Alzheimer’s disease. J Neurosci Res.

[52] Ahmed-Farid OAH, Ahmed RF, Saleh DO (2016). Combination of resveratrol and fluoxetine in an acute model of depression in mice: prevention of oxidative DNA fragmentation and monoamines degradation. J Appl Pharm Sci.

[53] Bagnall-Moreau C, Chaudhry S, Salas-Ramirez K, Ahles T, Hubbard K (2019). Chemotherapy-induced cognitive impairment is associated with increased inflammation and oxidative damage in the hippocampus. Mol Neurobiol.

[54] Badary OA, Sharaby SM, Kenawy SAE-B, El-Denshary EE-D, Hamada FMA (2000). Evaluation of cisplatin combined with ondansetron in Ehrlich ascites carcinoma in vitro and in vivo. Tumori J.

[55] Zirak MR, Rahimian R, Ghazi-Khansari M, Abbasi A, Razmi A, Ejtemaei Mehr S (2014). Tropisetron attenuates cisplatin-induced nephrotoxicity in mice. Eur J Pharmacol.

[56] Li Q, Guo D, Dong Z, Zhang W, Zhang L, Huang S-M (2013). Ondansetron can enhance cisplatin-induced nephrotoxicity via inhibition of multiple toxin and extrusion proteins (MATEs). Toxicol Appl Pharmacol.

[57] Kou W, Qin H, Hanif S, Wu X (2018). Nephrotoxicity evaluation on cisplatin combined with 5-HT 3 receptor antagonists: a retrospective study. Biomed Res Int.

[58] Keller L-A, Merkel O, Popp A (2022). Intranasal drug delivery: opportunities and toxicologic challenges during drug development. Drug Deliv Transl Res.

[59] Kumar M, Kakkar V, Mishra AK, Chuttani K, Kaur IP (2014). Intranasal delivery of streptomycin sulfate (STRS) loaded solid lipid nanoparticles to brain and blood. Int J Pharm.

[60] Uchida M, Katoh T, Mori M, Maeno T, Ohtake K, Kobayashi J (2011). Intranasal administration of milnacipran in rats: evaluation of the transport of drugs to the systemic circulation and central nervous system and the pharmacological effect. Biol Pharm Bull.

[61] Battaglia L, Panciani PP, Muntoni E, Capucchio MT, Biasibetti E, De Bonis P (2018). Lipid nanoparticles for intranasal administration: application to nose-to-brain delivery. Expert Opin Drug Deliv.

[62] Khan AR, Liu M, Khan MW, Zhai G (2017). Progress in brain targeting drug delivery system by nasal route. J Control Release.

