# Supplementary figures and images for: Intranasal ondansetron microemulsion counteracting the adverse effects of cisplatin: animal study
Source: Pharmacol Rep. 2022 Dec 14;75(1):199–210. doi: 10.1007/s43440-022-00435-3 (PMC9889460; doi:10.1007/s43440-022-00435-3)

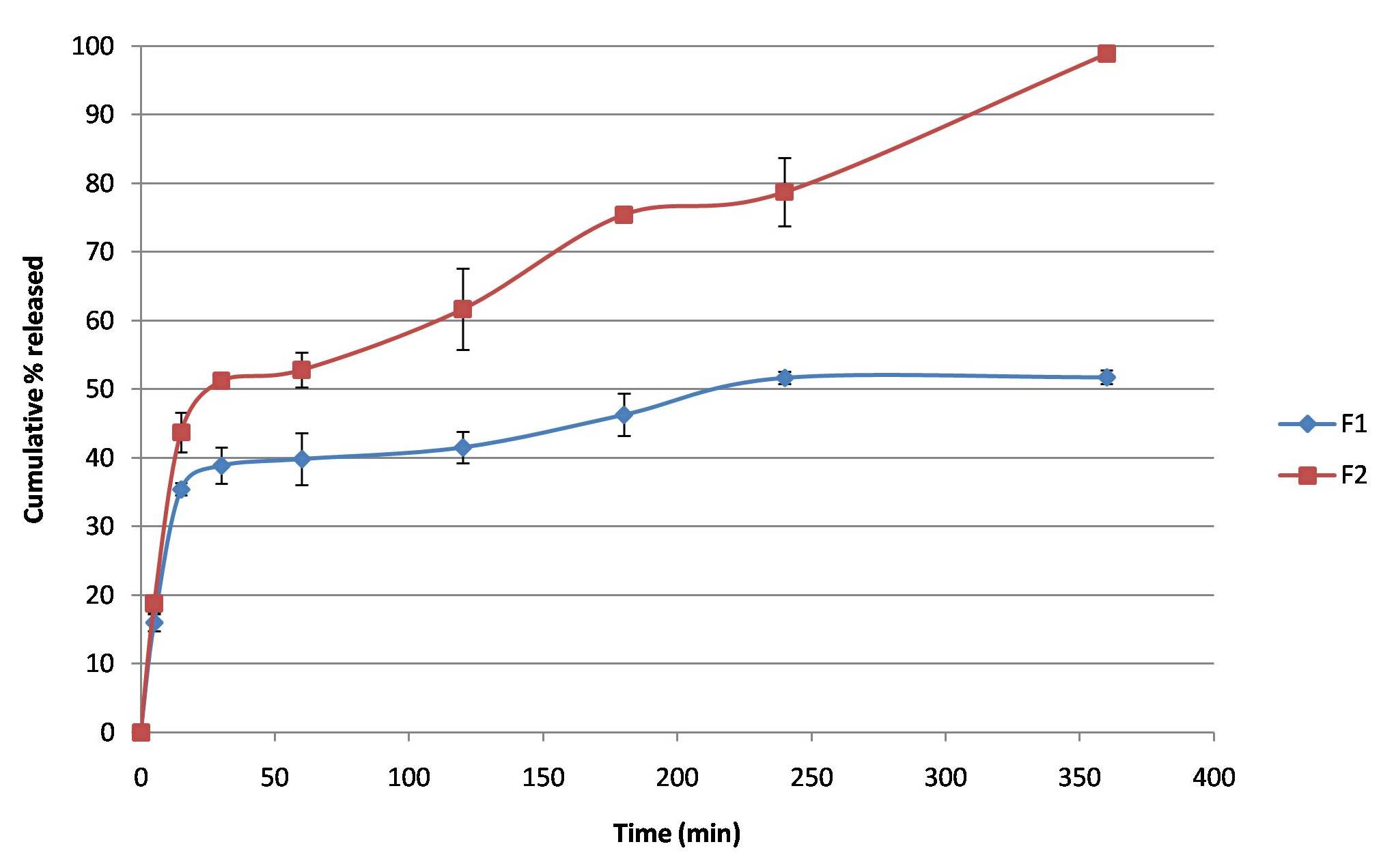

Supplement: Supplementary file 1 — Supplementary file1 (JPG 126 KB) [file 43440_2022_435_MOESM1_ESM.jpg]
