# Supplementary material for: Intranasal ondansetron microemulsion counteracting the adverse effects of cisplatin: animal study
Source: Pharmacol Rep. 2022 Dec 14;75(1):199–210. doi: 10.1007/s43440-022-00435-3 (PMC9889460; doi:10.1007/s43440-022-00435-3)
Supplement: Supplementary file 2 — Supplementary file2 (DOCX 1206 KB) [file 43440_2022_435_MOESM2_ESM.docx]

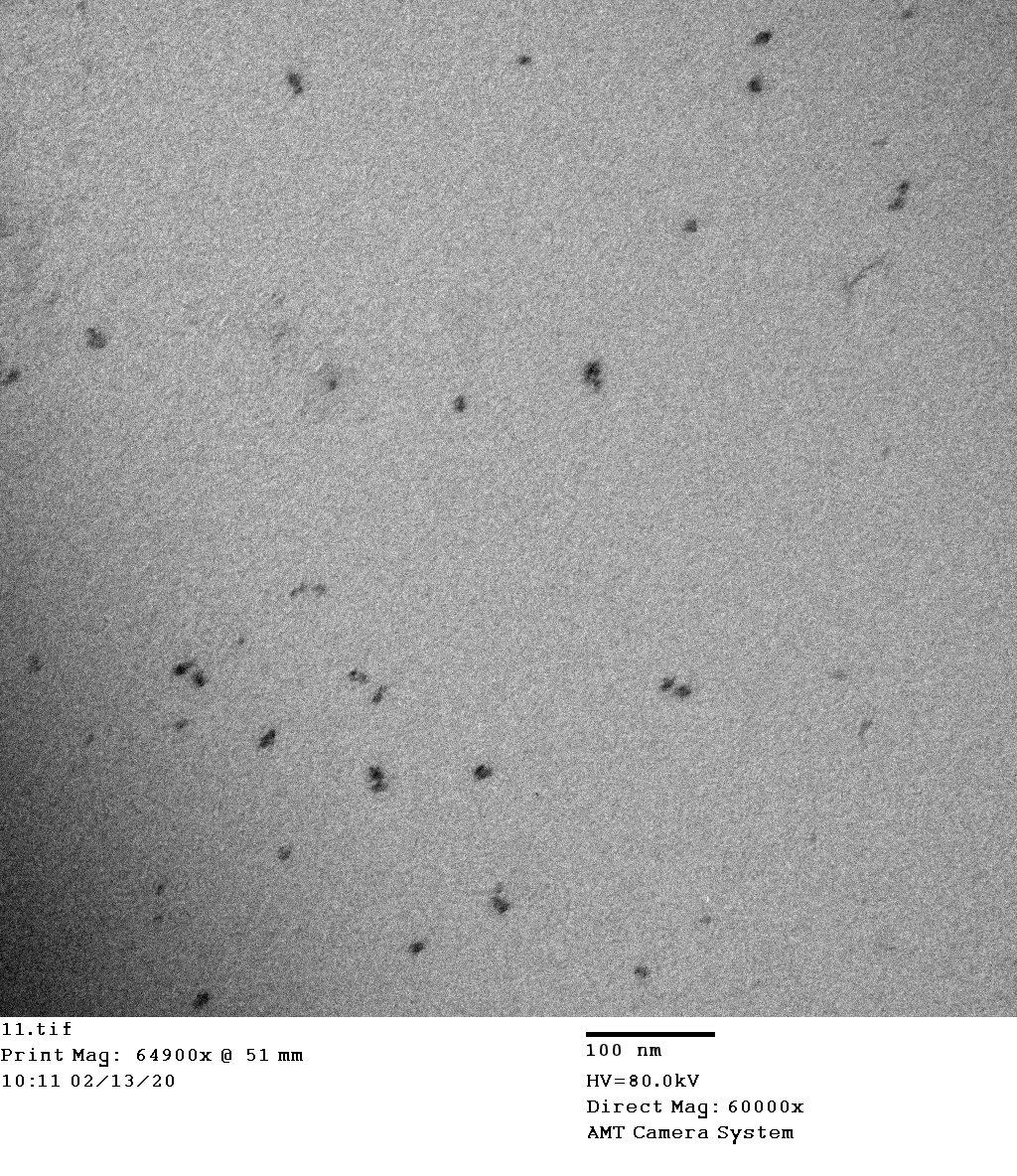


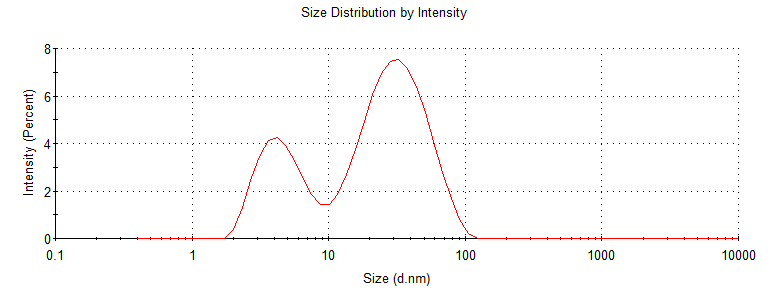


TEM microscopic image and particle size distribution (calculated by intensity percent) for formulation F2.
